# Searching for Peptide Inhibitors of T Regulatory Cell Activity by Targeting Specific Domains of FOXP3 Transcription Factor

**DOI:** 10.3390/biomedicines9020197

**Published:** 2021-02-17

**Authors:** Teresa Lozano, Noelia Casares, Celia Martil-Otal, Blanca Anega, Marta Gorraiz, Jonathan Parker, Marta Ruiz, Virginia Belsúe, Antonio Pineda-Lucena, Julen Oyarzabal, Juan José Lasarte

**Affiliations:** 1 Immunology and Immunotherapy, Center for Applied Medical Research (CIMA), University of Navarra, 31008 Pamplona, Spain; ncasares@unav.es (N.C.); cmartin.11@alumni.unav.es (C.M.-O.); blancaanega@gmail.com (B.A.); mgorraiz@unav.es (M.G.); jonathan.a.parker@kcl.ac.uk (J.P.); mruiz@unav.es (M.R.); virginiabelsue@hotmail.com (V.B.); 2 Molecular Therapeutics Programs, Center for Applied Medical Research (CIMA), University of Navarra, 31008 Pamplona, Spain; apinedal@unav.es (A.P.-L.); julenoyarzabal@external.unav.es (J.O.)

**Keywords:** T regulatory cells, Foxp3 transcription factor, immunotherapy of cancer, synthetic peptides, inhibition of protein–protein interaction

## Abstract

(1) Background: The ability of cancer cells to evade the immune system is due in part to their capacity to induce and recruit T regulatory cells (Tregs) to the tumor microenvironment. Strategies proposed to improve antitumor immunity by depleting Tregs generally lack specificity and raise the possibility of autoimmunity. Therefore, we propose to control Tregs by their functional inactivation rather than depletion. Tregs are characterized by the expression of the Forkhead box protein 3 (FOXP3) transcription factor, which is considered their “master regulator”. Its interaction with DNA is assisted primarily by its interaction with other proteins in the so-called “Foxp3 interactome”, which elicits much of the characteristic Treg cell transcriptional signature. We speculated that the disruption of such a protein complex by using synthetic peptides able to bind Foxp3 might have an impact on the functionality of Treg cells and thus have a therapeutic potential in cancer treatment. (2) Methods: By using a phage-displayed peptide library, or short synthetic peptides encompassing Foxp3 fragments, or by studying the crystal structure of the Foxp3:NFAT complex, we have identified a series of peptides that are able to bind Foxp3 and inhibit Treg activity. (3) Results: We identified some peptides encompassing fragments of the leuzin zipper or the C terminal domain of Foxp3 with the capacity to inhibit Treg activity in vitro. The acetylation/amidation of linear peptides, head-to-tail cyclization, the incorporation of non-natural aminoacids, or the incorporation of cell-penetrating peptide motifs increased in some cases the Foxp3 binding capacity and Treg inhibitory activity of the identified peptides. Some of them have shown antitumoral activity in vivo. (4) Conclusions: Synthetic peptides constitute an alternative to inhibit Foxp3 protein–protein interactions intracellularly and impair Treg immunosuppressive activity. These peptides might be considered as potential hit compounds on the design of new immunotherapeutic approaches against cancer.

## 1. Introduction

Cancer progression occurs when the immune system cannot constrain tumor growth. The ability of cancer cells to evade the immunoprotective network is due in part to their capacity to subvert the immune response by the induction and recruitment of T regulatory cells (Tregs) into the tumor microenvironment. In fact, Treg are generally regarded as an obstacle to the successful clinical application of tumor immunotherapy [1,2,3,4,5]. They are characterized by the expression of CD25 and the Treg-specific FOXP3 transcription factor, which is required for their development and function [6,7,8]. These cells can inhibit activation of other T-cells [9] and are needed for protection against autoimmune diseases and against graft versus host reactions after an organ transplant. However, the immunoregulatory function of Treg may hinder the induction of immune responses against cancer. Indeed, Tregs capable of suppressing the in vitro function of tumor-reactive T-cells have been found in humans in many types of tumors and have been associated with a high death hazard and reduced survival [1,3]. These studies support the notion that targeting Treg cells, either by depletion or functional modulation, may offer a significant therapeutic benefit, particularly in combination with other immune modulatory interventions such as vaccines and checkpoint blockade [10,11,12,13,14,15]. 

The FOXP3 transcription factor is essential for the programming and maintenance of Treg cells, and thus, it was considered as the “master regulator” of Treg cells [6,7,8]. The capacity of FOXP3 to bind DNA is critical for its functionality; however, it is clear that FOXP3–DNA interaction is assisted by FOXP3 cofactors and by multimerization [16,17]. The proteomic analysis of FOXP3 complexes showed that the majority of FOXP3 partners were proteins implicated in the regulation of transcription, including many sequence-specific transcription factors. It has been postulated that Treg cell phenotype and function is determined by the collective activity of this transcriptional network [17,18,19]. This hypothesis has important implications on the functionality of Tregs, and it raises new possibilities for the design of new therapies where Treg cells or FOXP3 expression have pathological consequences. It is clear that those compounds able to bind FOXP3 and inhibit or modify the FOXP3 interactome might have important consequences on the whole transcriptome signature of the FOXP3-expressing cell and consequently, on its activity. 

Historically, peptides have not been considered as drug candidates even though there are thousands of naturally occurring peptides with crucial roles in human physiology. So far, applications of chemically synthesized peptides have been importantly limited by their low systemic stability, poor specific biodistribution, high clearance, and poor membrane permeability [20,21]. However, there is an increasing interest in their use in therapy because of their high selectivity, efficacy, and tolerability (reviewed in [22]). In this work, we have undertaken the search for possible inhibitors of Foxp3 functions using different strategies. First, using a phage-displayed random peptide library, we identified 20 peptides able to bind with high affinity to FOXP3. Among them, we discovered P60, which is a peptide that is able to enter into the cells, bind to Foxp3, and impair its functions in vivo and in vitro [11,23]. Second, we synthesized short synthetic peptides encompassing different regions of FOXP3 with the aim to generate decoy molecules that are able to compete with FOXP3 in the generation of its protein interactome. In some of these identified peptides, we introduced chemical modifications such as N-term acetylation or C-term amidation, cyclizations, or the introduction of D-aminoacids and evaluated their effect of their inhibitory activity. This work has allowed us to identify potential hits for future development of Treg inhibitors for their use in immunotherapeutic approaches against cancer.

## 2. Experimental Section

### 2.1. Production of Recombinant Proteins

FOXP3, NFAT (Nuclear Factor Activated T cells), and AML1 (Acute Myeloid Leukemia 1 Protein) proteins were produced as described in previous works [11,12,23]. Briefly, plasmids pET20b-FOXP3, pET20b-AML1, and pET20b-NFAT were transfected into Escherichia coli BL21 bacteria competent for the expression and subsequent purification of the protein. The bacterial pellet was lysed in a French press (Thermo Electron Corporation), and protein purification was carried out from the resulted supernantant (for AML1 and NFAT) or from the inclusion bodies (FOXP3) by means of affinity chromatography using Histrap affinity columns and the platform of an FPLC. For alpha screen assays of FOXP3/AML1 interaction, a FOXP3-GST fusion protein was produced in Escherichia coli BL21 bacteria transfected with plasmid pDEST15-FOXP3 (provided by Dr. Casal, Centro Nacional de Investigaciones Oncológicas, Madrid, Spain) and purified as described previously [11].

### 2.2. Screening of FOXP3 Binder Peptides by Using Phage-Displayed Peptide Library and Biopanning

The technique associated to phage libraries has been used to initially identify peptides with the capacity to bind to FOXP3 by biopanning as previously described [24] with some modifications [11]. The phage library used contained 2 × 10^8^ different clones and was provided by the laboratory of George P. Smith (Division of Biological Sciences Tucker Hall, University of Missouri, USA). The biopanning process was carried out by three binding/elution rounds, each time with a lower amount of FOXP3 adhered to the wells (being progressively reduced in each round from 2.5 to 0.02 Pg/mL, and finally to 0.002 Pg/mL). After the last round, colonies of bacteria infected by a single phage were grown, DNA were isolated, and the portion of the genome including the region corresponding to the peptides displayed in the pIII protein of the phage was sequenced. To restrict the number of peptides to be assayed, a commercial ELISA was carried out, based on an anti-M13 monoclonal antibody of the phage (HRP/Anti-M13, Amersham Pharmacia Biotech), for the purpose of only selecting the phages with higher affinity for FOXP3.

### 2.3. Peptide Synthesis

Peptides were synthesized using the Apex 396 Automated Multiple Peptide Synthesizer (Aapptec, Kentucky, VA, US) by the solid phase method of Merrifield using the fluorenylmethyloxycarbonyl alternative as previously described [25]. Cyclic peptides were synthesized by Wuxi AppTech (Shanghai, China). The purity of peptides was 90% as judged by HPLC. Sequences of peptides derived from the phage-displayed peptide library screening campaign are shown in Table 1. Overlapping peptides derived from the forkhead (FKH) domain from FOXP3 are shown in Table 2. They have been named using numbers corresponding to their location within the FOXP3 sequence (accession number NP_054728.2). In some experiments, peptide 254–267 from ovalbumin (OVA) was used as a control peptide (Ctrl pept).

### 2.4. Biomolecular Interaction Analysis by Surface Plasmon Resonance and by Alphascreen Technology

The screening of peptide binders to FOXP3 was performed by surface plasmon resonance (SPR) using a ProteOn XPR36 (Bio-Rad, Hercules CA, USA) optical biosensor. Recombinant protein FOXP3-6His produced and purified from E-coli was immobilized covalently onto a GLC sensor chip (Bio-Rad) using the coupling reagents sulfo-NHS (N-Hydroxysulfosuccinimide) and EDC (1-Ethyl-3-(3-dimethylaminopropyl) carbodiimide, (Bio-Rad) as previously described [23]. Peptide solutions (10 μM) were injected by triplicate in running buffer (phosphate-buffered saline, 0.005% (*v*/*v*) Tween 20, pH 7.4) at a flow of 30 μL/min. The interspot signal (obtained in the chip surface not immobilized with protein) was used as reference. FOXP3/NFAT1 and FOXP3/AML1 interactions were analyzed by Alphascreen technology as previously described [23] according to the manufacturer’s protocol (Perkin Elmer, Benelux). Reactions were performed in a 40 μL final volume in 96-well Optiwell microtiter plates (Perkin Elmer). The reaction buffer contained 20 mM HEPES (4-(2-hydroxyethyl)-1-piperazineethanesulfonic acid), pH 7.9, 200 mM KCl, 1 mM MgCl2 and 0.05% bovine serum albumin (BSA). Recombinant NFAT1 expressed with a hexa-Histidine tag was captured by nickel chelate acceptor beads, whereas recombinant FOXP3 expressed with a GST (glutathione S-transferase) tag was captured by glutathione donor beads (Perkin Elmer). For FOXP3 dimerization assays, GST-tagged FOXP3 and His-tagged FOXP3 were co-cultured in the presence or absence of the indicated peptides for 1 h. Then, donor and acceptor beads were added as described above and incubated for 2 h. FOXP3/AML1 interaction was measured following a similar protocol using FOXP3-GST and AML1-His proteins. Exposure of the reaction to direct light was avoided as much as possible, and the emission of light from the acceptor beads was measured in the EnVision plate reader after the indicated incubation period (Perkin Elmer, Benelux).

### 2.5. Co-Immunoprecipitation and Western Blot Analysis 

HEK293 cells were cultured in Dulbecco modified Eagle medium (Invitrogen) supplemented with 8% heat-inactivated fetal calf serum (FCS), penicillin, and streptomycin (Invitrogen) at 37 °C and 5% CO2. Plasmids cells were grown to 50% confluence in 6-well plates (Nunc) and transfected with a mixture of 2 μg of DNA plasmids (1 μg of FLAG-FOXP3 (expressing Foxp3 linked to the DYKDDDDK peptide tag and 1 μg of HA-NFAT expressing NFAT linked to the YPYDVPDYA peptide tag), kindly provided by Dr Paul, Coffer, Utrech, The Netherlands)) and 8 μL PEI (polyethylenimine) overnight; the next day, cells were treated with 393–403 L, 397–406 C, or an irrelevant peptide at 100 μM. Twenty four hour later, HEK 293 cells were lysed in NP40 lysis buffer (0.05 M Tris-HCl pH 7.5, 0.5% Nonidet P40, 0.15 M NaCl, 0.01 M EDTA) containing a HALT^TM^ protease inhibitor cocktail (ThermoScientific), and immunoprecipitation was performed utilizing anti-FLAG coupled beads (Sigma) during 2 h at 4 C. Beads were washed 3x in lysis buffer, boiled, and samples were separated by SDS-PAGE, electrophoretically transferred to polyvinylidene difluoride membrane (Millipore, Bedford, MA), and hybridized with antibodies as indicated. Immunocomplexes were detected using enhanced chemiluminescence (Biorad).

### 2.6. In Vitro Assays for Treg Inhibition

In vitro assays were carried out using (i) the Karpas 299 human cell line (ACC-31, DSMZ, Germany), derived from a human lymphoma with a regulatory T cell profile [26] or (ii) using natural Treg cells isolated from the murine splenocytes. To evaluate the effect of peptides on the immunosuppressive activity of the Karpas 299 cell line, a “mixed lymphocyte reaction” (MLR) assay was carried out using peripheral blood mononuclear cells (PBMCs) from two donors (previously tested to induce a strong MLR reaction in co-culture) (1 × 10^5^ cells/well per each PBMC) in the presence or absence of Karpas 299 (1 × 10^4^ cells/well). Peptides were added at 50 μM to the co-cultures to evaluate their capacity to restore the production of interferon-gamma (IFN-γ) inhibited by Karpas 299. After 48 hours, the supernatants were extracted to measure interferon-gamma (IFN-γ) by means of a commercial ELISA (Pharmingen, San Diego, CA, US). *In vitro assays for Treg inhibition using natural Treg cells:* Murine CD4+CD25+ (Treg cells), and CD4+CD25-T-cells (effector T cells) were purified from murine spleen cells by using a murine regulatory T-cell isolation kit (Miltenyi Biotec, Bergisch Gladbach, Germany) according to the manufacturer’s instructions. The purity of the resulting T-cell populations was confirmed to be >95% by flow cytometry. Inhibition of murine T regulatory cell function was measured in an in vitro assay of T-cell stimulation. Effector T cells (10^5^ cells/well) from BALB/c mice were stimulated in vitro with 2, 5 μg/mL of anti-mouse CD3 antibody (Pharmingen) in the presence/absence of purified Treg cells (10^4^ cells/well) and the indicated peptides (50 μM). T-cell proliferation was measured 3 days later as previously described [23]. Percentage of Treg inhibition was calculated using the following formula: % inhibition= 100*((cpm of Teff&Treg co-cultures in the presence of peptide - cpm of Teff&Treg co-cultures)/(cpm of Teff - cpm of Teff&Treg co-cultures)). The Institutional Review Board on Human Subjects (Clínica Universidad de Navarra, Ref 2016.118) approved this research, and informed consent was obtained from all blood donors.

### 2.7. Tumor Cell Lines

The cell line 4T1-FOXP3 expressing FOXP3 was generated. 4T1-WT cells were transfected with empty pcDNA3.1 or with pcDNA3.1 FOXP3, expressing FOXP3 gene and neomycin (which confers resistance to G418 antibiotic) to generate 4T1-Ctrl and 4T1-Foxp3 cells. Briefly, 8 × 10^5^ cells were seeded in 6-well culture plates one day prior to transfection. The cultures were 60–80% confluent at the time of transfection. Cells were transfected with 5 ug plasmid DNA per well for 6 hours using lipofectimine 2000 transfection reagent (Invitrogen). After 48 hours, 0.3 mg/mL of G418 drug (GIBCO) was added to the culture during 14 days for the selection of resistant cells. These cells were used in vitro to measure the effect of peptides to overcome the proliferative capacity inhibited by FOXP3. In addition, 4T1-FOXP3 and 4T1-Ctrl cells were injected in vivo (10^5^ cells/ mouse) subcutaneously or intravenously to compare their capacity to induce tumors and lung metastases. Murine Lewis lung carcinoma expressing OVA, LLCOVA were kindly provided by Dr. Daniel Ajona (CIMA). The murine colon adenocarcinoma cell line MC38, LLOOVA, 4T1-Ctrl, and 4T1-FOXP3 were cultured in mouse medium (RPMI 1640, 10% fetal calf serum (Sigma), 100 U/mL penicillin, 100 μg/mL streptomycin (Invitrogene), 10 mg/mL Gentamicin (Gibco), 2 mM L-Glutamine (Lifetechnologies), 5 mM β-mercaptoethanol (Sigma), and fungizone (GIBCO). All cell lines were cultured at 37 °C in a humidified atmosphere with 6.5% CO2.

### 2.8. In Vivo Tumor Experiments

MC38 cells (5 × 10^5^ cells/mouse), LLCOVA (1.5 × 10^6^ cells/mouse), or TC1 (P3A15) cells (5 × 10^5^ cells/mouse), were injected subcutaneously (sc) in C57/BL6 mice (n = 8 mice per experimental group). Ten days later, when the tumor reached 5 mm in diameter, mice were randomly divided into different experimental groups. A group of mice were treated intraperitoneally (i.p.) with the indicated peptide (one dose of 50 μg/mouse per day during 10 consecutive days). Tumor volume in mm^3^, calculated using the formula V = (length × width^2^)/2, was measured at regular intervals. Mice were sacrificed when tumor size reached a volume greater than 4 cm^3^. *Immune cells characterization in LLCOVA tumor model.* Mice bearing LLCOVA tumors and treated with the peptides were sacrificed three day after the last peptide administration. Spleens and tumors were collected and processed for analysis. *ELISPOT*: 8*10^6^ splenocytes were stimulated with SIINFEKL peptide (1 μg/mL) for 16 hours. Numbers of IFN-γ spots were measured by the ELISPOT technique (BD bioscience) as previously described [23]. *Antibodies and flow cytometry.* The following fluorochrome-conjugated antibodies to surface and intracellular antigens (BD Biosciences) were used at 0.25–1 μg/mL: CD8α (53-6.7), CD4, (RM4-5), CD45.2 (104), and NKP46 (29A1.4). Cells were incubated with a Zombie NIR™ Fixable Viability kit (Biolegend) for 15 min at room temperature and then washed once with washing buffer (PBS without Ca/Mg, 0.5 M EDTA, 10% of fetal bovine serum and 1% penicillin/streptomycin). Subsequently, cells were incubated with specific antibodies for 30 min on ice in the presence of 2.4G2 monoclonal antibody (mAb) to block unspecific FcγR binding. Cells were fixed and permeabilized with the FOXP3/Transcription Factor Staining kit buffers (ebioscences) and then stained intracellularly (15 min at room temperature) with fluorochrome-conjugated mAbs against mouse, IFN-γ (XMG1.2) and FOXP3 (FJK-16s). Data acquisition was performed with a FACS Canto II flow cytometer (Becton Dickinson) and analyzed by FlowJo software (TreeStar). Mice were housed in appropriated animal care facilities during the experimental period and handled following the international guidelines required for experimentation with animals. An institutional ethical committee approved the experiments (Refs R-131-16GN and R-018-19GN).

### 2.9. Statistics

Normality was assessed with the Shapiro–Wilk W test. Statistical analyses were performed using parametric (Student’s t test and one-way ANOVA) and non-parametric (Mann–Whitney U and Kruskal–Wallis) tests. For all tests, a *p* value < 0.05 was considered statistically significant. Descriptive data for continuous variables are reported as means ± SEM. GraphPad Prism for Windows was used for statistical analysis.

## 3. Results

### 3.1. Screening of FOXP3 Inhibitors by Using Phage-Displayed Peptide Library

**Figure 1:**
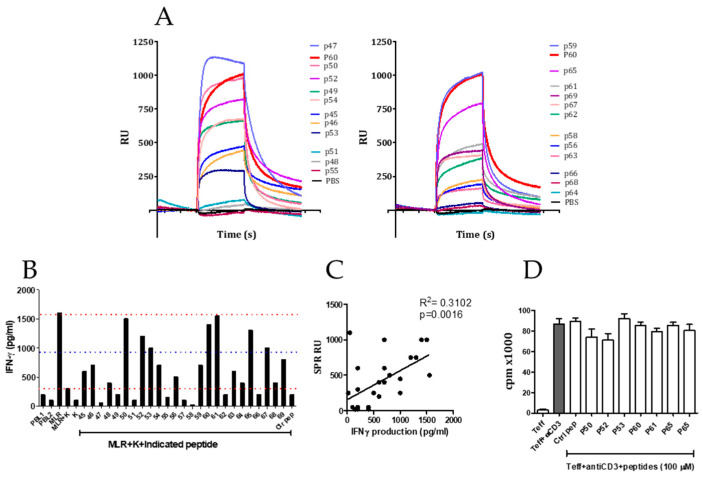
Screening of FOXP3 inhibitors. (**A**) FOXP3 binder peptides identified using phage-displayed peptide library were tested on their capacity to bind FOXP3 in surface plasmon resonance (SPR) using a chip coated with FOXP3 protein. (**B**) Capacity of the peptides to restore the interferon-gamma (IFN-γ) production of a mixed leukocyte reaction (MLR) co-cultured with the Karpas (K) T regulatory cell (Treg) cell line. (**C**) Correlation between FOXP3 binding capacity of the peptides (measured in (**A**) by SPR) and their Treg inhibitory capacity measured in (**B**). (**D**) Effect of selected peptides on T cell proliferation of effector murine T cells in response to anti-CD3 antibody stimulation.

For the purpose of obtaining peptides capable of binding to FOXP3 with high affinity, an M13-based phage library expressing 15-mer peptides fused to the N-terminal end of the pIII coat protein was used. The screening process was carried out by means of an affinity assay or biopanning (see Methods). After the third round of selection, a total of 100 bacterial clones (infected with unique phages expressing peptides able to bind to FOXP3) were obtained. To reduce the number of peptides to be assayed, a commercial ELISA based on an anti-M13 monoclonal antibody of the phage was carried out to select the phages with higher affinity for FOXP3 (not shown). After this second filter, 47 phage-infected bacteria clones were grown to isolate the DNA for sequencing the portion of the genome corresponding to the peptides displayed in the pIII protein of the phage. Sequencing analyses allowed us to identify 20 fifteen-mer peptides that were synthesized and tested on their capacity to bind FOXP3 coated to a chip by surface plasmon resonance (Table 1). Figure 1A shows the results obtained by this screening. Peptides were classified in two categories according to their capacity to bind FOXP3 coated to the chips by SPR. Thus, peptides p47, p59, p60, p50, p52, p65, p49, and p55 were considered strong FOXP3 binders (>500 RU). 

After this first biochemical assay, we then evaluated these peptides in a cell-based assay. Thus, their capacity to inhibit the immunosuppressive activity of the regulatory T cell line Karpas [26,27,28] in a mixed leukocyte reaction (MLR) was tested. PBMCs from two different healthy donors (PBMC1 and PBMC2) were mixed in culture to stimulate the production of IFN-γ. The IFN-γ production is significantly inhibited if the Treg cell line Karpas is added to the cell culture. In this setting, individual peptides were added to see if cytokine production inhibited by Karpas cell line was restored by the presence of the peptide. After 48 h of cell co-culture, we measured the IFN-γ released to the supernatant by ELISA. As shown in Figure 1B, the addition of Karpas cells to these cultures strongly inhibited the production of IFN-γ. However, notably, seven peptides out of the total 20 were able to restore in more than 50% the IFN-γ production inhibited by the addition of the Karpas cell line. 

As opposed to a cell-free biochemical assay such as the SPR, evaluation of the inhibitory activity of FOXP3 peptide binders in a cell-based assay implies that peptides must enter into the cell crossing the cell membrane, which is a limitation for the activity of peptide inhibitors of intracellular targets [20]. It could happen that some good binders might not be able to cross the cell membrane. Moreover, binding capacity might not necessarily mean an inhibitory activity of FOXP3 function. However, we found a significant correlation between the capacity of peptides to bind FOXP3 and its ability to restore IFN-γ production (*p* = 0.0016, Figure 1C). In these assays, we identified seven peptides (p50, p52, p53, p60, p61, and p65) with potential Treg inhibitory capacity. In a different experiment, we evaluated the effect of these seven peptides on the proliferation of T cells alone in response to anti-CD3 stimulation during 48 h to discard a potential direct effect on effector T cells. No significant changes on T cell proliferation were observed in this assay (Figure 1D). The results obtained with the Karpas cell line should be taken with caution, since there is not a formal demonstration of the role of Foxp3 in the inhibitory activity of the Karpas cell line. 

**Figure 2:**
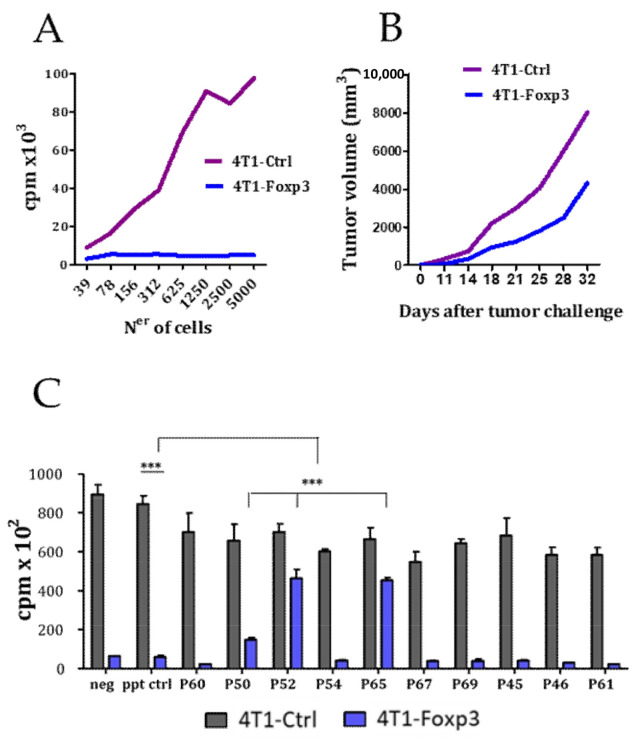
Effect of synthetic peptides on the cancer-suppressor activity of FOXP3. Effect of ectopic expression of FOXP3 in 4T1 cell line on cell proliferation in vitro (**A**) and on in vivo tumor growth in mice (n = 6 mice per group) (**B**). (**C**) Effect of peptides (50 μM) on the proliferation of 4T1-Ctrl and the 4T1-FOXP3 expressing tumor cell line in vitro. Data are representative of two independently repeated experiments. *** *p* < 0.005. One-way ANOVA with Bonferroni multiple comparison test. Bars and error show mean and SEM.

FOXP3 expression is not restricted to the lymphocyte lineage, but it is also present in some cancer cells, especially in breast cancer cells, where it has been demonstrated to be a cancer-suppressor gene and an important regulator of the HER2/ErbB2 and SKP2 oncogenes [29]. FOXP3 has various distinguishable functional domains: (i) an N-terminal domain (from a.a. 1 to 193) responsible for transcriptional repression, (ii) a zinc finger (a.a. 200–223), a leucine-zipper (LZ)-like motif (a.a. 240–261), which facilitates the formation of FOXP3 homo-dimers or tetramers, and (iii) the highly conserved carboxy terminal forkhead domain (FKH; from a.a. 338 to 421) responsible for the DNA binding [30]. It has been described that the tumor suppressor activity of FOXP3 is located in the N-terminal region of the protein (Aa 1-196) [31]. In a recent work, Gong et al. have shown that the ectopic expression of FOXP3 inhibited hepatocarcinoma cell line (HCC) proliferation, migration, and invasion, while FOXP3 downregulation promoted HCC growth [32]. To evaluate the antitumoral effect of FOXP3, we transfected the 4T1 tumor cells with the plasmid pcDNA-FOXP3 for the stable expression of FOXP3. We found that FOXP3 expression impaired tumor cell growth in vitro (Figure 2A) as well as in vivo when the cells were injected subcutaneously into BALB/c mice (Figure 2B). Since the 20 peptides identified from the biopanning assay using the phage-displayed peptide library could bind to any part of FOXP3, it could be postulated that some of them might have an impact on the tumor-suppressor activity of FOXP3. Thus, we evaluated the activity of a panel of these peptides on the proliferative capacity of 4T1-Ctrl and 4T-1 FOXP3 cells in vitro. Interestingly, it was found that peptides p50, p52, and p65, in addition to their Treg inhibitory capacity, were also able to inhibit the tumor-suppressor activity (Figure 2C). 

### 3.2. Screening of Treg Inhibitors by Using Peptides Encompassing the Leucine Zipper Domain of FOXP3 

**Figure 3:**
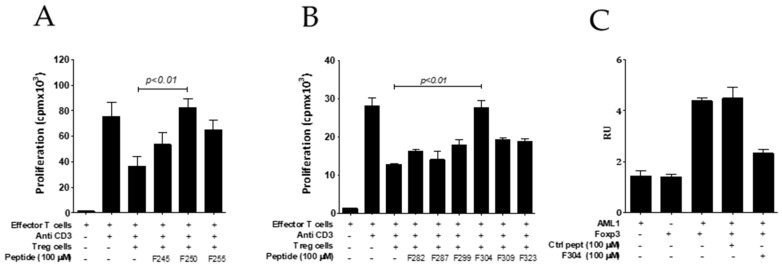
Screening of FOXP3 inhibition by peptides derived from the leucine zipper (**A**) and AML1 (**B**) binding domains. Effector T cells (CD4+CD25−) isolated from the spleen of BALB/c mice were stimulated with anti-CD3 in the presence or absence of purified murine CD4+CD25+ Treg cells (Tregs) and the indicated peptide (50 μM). Three days later, cell proliferation was analyzed by measuring tritiated thymidine incorporation. Data are representative of two independent experiments. One-way ANOVA with Bonferroni multiple comparison test. (**C**) Disruption of FOXP3-AML1 heterodimerization by peptide F304–318 measured by alphascreen. Bars and error show mean and SEM.

It has been described that leucine zipper domain of FOXP3 is required for FOXP3 homodimerization [30,33] and the suppressive function of Tregs [34]. For these reasons, we synthesized 15-mer peptides encompassing aminoacids 245–260, 250–265, and 255–270 from the LZ domain to evaluate their potential inhibitory activity on Treg cell function in vitro. Interestingly, it was found that peptide p250–265 was able to fully restore the proliferative capacity of effector T-cells stimulated with anti-CD3 antibody in the presence of Treg cells (Figure 3A). However, we could not demonstrate an inhibitory activity of this peptide on FOXP3 homodimerization (measured by alpha screen), and further experiments are needed to confirm the mechanism of action of this peptide.

### 3.3. Screening of Treg Inhibitors by Using Peptides Encompassing the AML/Runx1 Binding Domain of FOXP3

The region encompassed by aminoacids 278 and 336 from FOXP3 has been described to be implicated in its interaction with the transcription factor AML1/Runx1 protein [35]. AML1 is required for the activation of IL-2 (interleukin 2) and IFN-γ gene expression in conventional CD4+ T-cells. FOXP3 interaction with AML1 in Treg cells suppresses IL-2 and IFN-γ production, upregulates Treg-associated molecules, and plays a role in the Treg suppressive [35] activity. Consequently, the blocking of FOXP3/AML1 interaction might have an impact on Treg activity. Thus, we synthesized six 15-mer peptides encompassing this region (F282–296, F287–301, F299–313, F304–318, F309–323, and F323–337) that were tested on their capacity to inhibit Treg in vitro. Peptides sequences are shown in Table 2. We found that peptide F304–318 was able to inhibit Treg activity overcoming efffector T cell proliferation in response to anti-CD3 stimulation (Figure 3B). Interestingly, we found that peptide F304–318 was also able to impair FOXP3/AML1 heterodimerization (measured by alphascreen) (Figure 3C).

### 3.4. Screening of Treg Inhibitors Using Peptides Encompassing the FKH Domain of FOXP3

**Figure 4:**
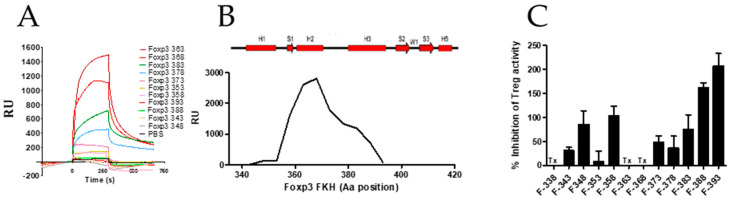
Screening of FOXP3 inhibitors with peptides from the FOXP3 forkhead (FKH) domain. (**A**) Binding capacity of the synthetic peptides to a chip coated with FOXP3 measured by SPR. (**B**) Relative FOXP3 binding capacity of different regions from FKH to FOXP3 deduced by the SPR analysis with 15-mer overlapping peptides. Those in red represent the structural elements presented in the FKH domain according to the crystal structure [37]. (**C**) Treg inhibitory capacity of FKH FOXP3-derived peptides. Effector T-cells (CD4+CD25− spleen cells) from BALB/c mice were stimulated with anti-CD3 in the presence or absence of purified murine CD4+CD25+ Treg cells (Treg) and the indicated peptide (50 μM). Three days later, cell proliferation was analyzed by measuring tritiated thymidine incorporation. Percentage of inhibition was calculated with respect to T-cell proliferation in the presence/absence of Treg cells. Values are mean ± SEM. Tx: toxic.

FOXP3 can regulate the gene expression of a number of genes that are also targets for the transcription factor NFAT, which, in cooperation with AP-1 (Fos/Jun), can activate many genes during lymphocyte activation [36]. This regulatory capacity of FOXP3 was justified by its ability to interact physically with NFAT and regulate its activity [37]. Therefore, the inhibition of these interactions might lead to the impairment of specific functions of FOXP3 and Treg activity and thus be beneficial in the development of vaccines and tumor therapies. We synthesized twelve 15-mer overlapping peptides encompassing the FKH domain of FOXP3 and evaluated their binding capacity to FOXP3 by SPR (Figure 4A). We identified peptides F363–377, F368–382, F378–392, and F383–397 as good binders to FOXP3. Peptide sequences are shown in Table 2. These peptides define an intermediate region within the FKH domain (Figure 4B) that includes the FOXP3 FKH dimerization interface [37]. Notably, two mutations in this interface, F371C and F373A, have been described in IPEX patients, which are characterized by a severe autoimmune syndrome (IPEX: immune dysregulation, polyendocrinopathy, enteropathy, X-linked) [38,39], suggesting that the inhibition of the FKH dimerization process may affect Treg activity. Therefore, we studied the capacity of these peptides to inhibit Treg activity in vitro at 50 μM. Peptides F348–362, F358–372, F388–402, and F393–407 showed a strong Treg inhibitory capacity, whereas peptides F363–377 and F368–382 seemed to have some toxicity at 50 μM (cpm below the cpm reached in Teff&Treg co-cultures in the absence of peptide) (Figure 4C). When these two peptides were tested at lower concentration (10 μM), no toxicity was observed; however, they did not exhibit Treg inhibitory capacity (not shown). Interestingly, we found that the C terminal part of the FKH domain, in particular peptide F393–407, displayed a strong Treg inhibitory capacity. Indeed, the level of T-cell proliferation obtained in the presence of this peptide even surpassed the maximum proliferation of T-cells stimulated with anti-CD3 in the absence of Treg cells. This effect could be ascribed to the transient expression of FOXP3 after T cell receptor (TCR) stimulation that might have a regulatory role limiting T-cell proliferation, as it has been suggested previously [11,12,40,41]. Based on the crystal structure of the FOXP3/NFAT/DNA complex [37], residues E399, E401, and K403 from the FKH domain are closely implicated in the formation of the cooperative complex between FOXP3 and NFAT, which is key for the suppression function of Treg cells. We found that peptide F393–403, a truncated version of peptide F393–407, inhibited FOXP3–NFAT interaction and improved effector T-cell functions in response to TCR stimulation and delayed tumor growth in a model of hepatocellular carcinoma [23]. These results suggest that the disruption of FOXP3:NFAT interaction with short synthetic peptides might have potential therapeutic applications in cancer. 

### 3.5. Peptide Modifications to Improve the Treg Inhibitory Capacity of Selected Peptides

**Figure 5:**
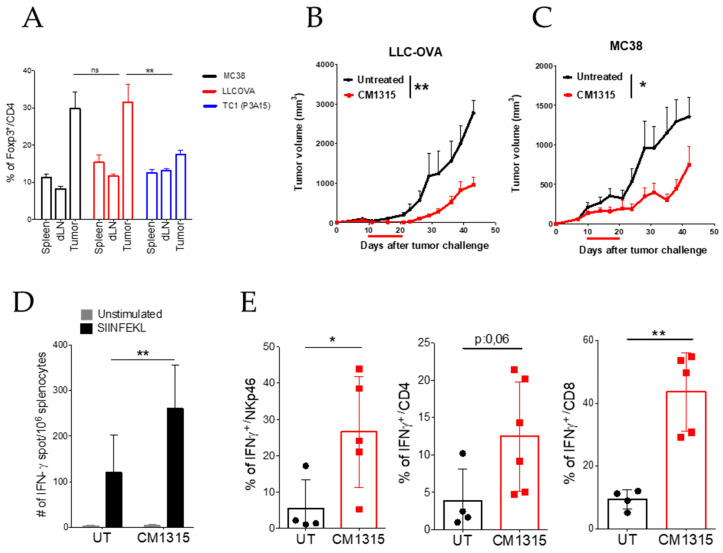
Antitumor activity of cyclic peptide CM1315. (**A**) Number of intratumor FOXP3+ cells in mice bearing MC38, LLCOVA (a lung cancer cell line expressing ovalbumin as a model tumor antigen), or TC1 (P3A15) tumors. (**B**,**C**) Antitumor effect of CM1315 administration in mice bearing LLCOVA (**B**) or MC38 (**C**) tumors (n = 8 mice per group). (**D**) Number of IFN-γ producing cells specific for the SIINFEKL peptide in the spleen of mice bearing LLCOVA tumors and treated with CM1315 or with saline. (**E**) Number of IFN-γ producing CD4, CD8, or NK cells infiltrating the tumor of mice treated with CM1315 or with saline. Data are representative of two independently repeated experiments. * *p* < 0.05, ** *p* < 0.01, one-way ANOVA with Bonferroni multiple comparison test (**A**), Log-rank test (**B**,**C**), two-way ANOVA with Bonferroni multiple comparison test (**D**), Student´s *t*-test (**E**). Bars and error show mean ± SEM.

From the screening campaign using a phage-displayed peptide library (Section 3.1), we identified the fifteen-mer peptide P60 as a peptide that is able to enter into the cells, bind FOXP3, and impair FOXP3 nuclear translocation and inhibit Treg activity in vitro and in vivo [11]. The P60 peptide was able to improve the antitumor effect of other immunotherapeutic approaches [42,43,44,45,46,47,48] constituting a potential candidate drug to inhibit Treg activity. An alanine scanning was conducted to identify key positions within the P60 sequence for FOXP3 binding P60. The introduction of double mutants, as well as a D-alanine at position 2 and a head-to-tail cyclization allowed us to identify molecule CM1315, which significantly improved the P60 half life and Treg inhibitory activity [23]. After this optimization, we have tested the antitumor activity of CM1315 in mice bearing tumors. We evaluated CM1315 in murine models of colon cancer (MC38 cells) and lung cancer (LLCOVA, a lung cancer cell line expressing ovalbumin as a model tumor antigen) because in both models, there is a high proportion of Treg cells infiltrating the tumor. Indeed, in both cases, almost 30% of CD4+ T-cells are FOXP3+ as compared to the normal levels of FOXP3+ Treg found in the spleen or in the tumor-draining lymph nodes (Figure 5A). Thus, mice were challenged with the MC38 colon cancer cell line or with the LLC-OVA lung tumor cell line, and 7 to 10 days later, when the tumor size reached 5 mm in diameter, they were treated with CM1315 (a single administration of 50 μg/mouse during ten consecutive days). We found in both models that peptide treatment was able to significantly delay tumor growth (Figure 5B,C). We took advantage of ovalbumin expression in the LLC-OVA tumor model to measure the antitumor immune response specific for the SIINFEKL peptide (the immunodominant cytotoxic T cell epitope (CTL epitope) within ovalbumin). We found a significant increase in the number of IFN-γ-producing cells specific for SIINFEKL (measured by ELISPOT) in the spleen in mice treated with CM1315 during 10 consecutive days (Figure 5D). Similarly, the number of tumor-infiltrating CD4+, CD8+, and NK (natural killer) cells able to produce IFN-γ was significantly higher in mice treated with CM1315 (Figure 5E), suggesting that Treg inhibition improved the antitumor immune response.

**Figure 6:**
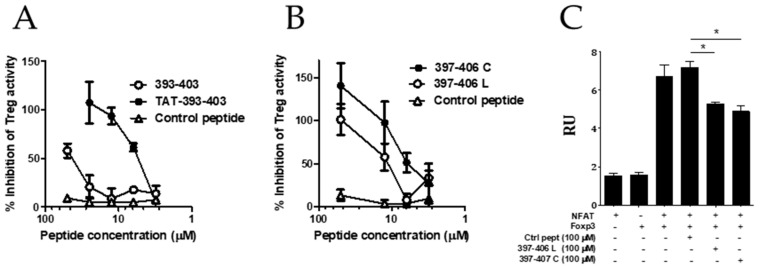
Peptide modifications to improve the Treg inhibitory capacity of the selected peptides. (**A**) The effect of fusion of peptide F393–403 to the TAT peptide or (**B**) a head-to-tail cyclization of the peptide F397–406 on the Treg inhibitory activity in vitro. Effector T-cells (CD4+CD25- spleen cells) from BALB/c mice were stimulated with anti-CD3 in the presence or absence of purified murine CD4+CD25+ Treg cells (Treg) and the indicated peptide (50 μM). Three days later, cell proliferation was analyzed by measuring tritiated thymidine incorporation. Percentage of inhibition was calculated with respect to T-cell proliferation in the presence/absence of Treg cells. (**C**) Disruption of FOXP3-NFAT heterodimerization by the linear and cyclic versions of peptide F397–406 (397–406 L and 397–406 C respectively) measured by alpha screen. Values are mean ± SEM. Data are representative of two independently repeated experiments. * *p* < 0.05, one-way ANOVA with Bonferroni multiple comparison test.

We have also focused on the screening campaign of peptides derived from the FOXP3 FKH domain (Section 3.3). Although we identified several peptides encompassing the intermediate region of FKH with high capacity to bind to FOXP3, we focused on peptide F393–407, based on its ability to inhibit Treg activity in vitro. The truncation of peptides at the C and N-terminus allowed us to identify the peptide F393–403 as the best linear version of the original peptide able to inhibit Treg activity in vitro. We found that peptide F393–403 was able to inhibit FOXP3:NFAT interaction and demonstrated that in vivo peptide administration had antitumor activity in a murine model of hepatocellular carcinoma [12]. Since the peptide had poor pharmacokinetics in vivo, we tried to introduce some modifications to its sequence. On the one hand, we tried to improve the cell permeability of the peptide by its fusion to the cell-penetrating peptide TAT (Tat 44–57 Sequence CGISYGRKKRRQRRR, which is known for its membrane translocation characteristics [45]). Thus, peptide TAT-393–403 was synthesized and compared with peptide 393–403 in the in vitro assay of Treg inhibition. It was found that fusion of the peptide to the cell-penetrating peptide TAT significantly improved the Treg inhibitory capacity (Figure 6A). 

On the other hand, we tried to reduce the size of the peptide and introduced a head-to-tail cyclization of the peptide to improve the cell permeability and to augment the half-life by protecting N and C terminal positions of the peptide and make the peptide more resistant to proteases. The crystal structure of the NFAT1:FOXP3:DNA complex revealed that aminoacids 397 and 406 are in close proximity, suggesting that a backbone cyclization of this peptide could stabilize the structure and even improve the affinity for NFAT binding. Thus, a macrocycle consisting of a head-to-tail cyclization of peptide 397–406 (397–406 C) was synthesized and tested for its activity to inhibit Treg activity in vitro. As shown in Figure 6B, head-to-tail cyclization improved the Treg inhibitory capacity as compared to the linear version (397–406 L) of the same peptide. Notably, both peptides retained the activity to impair Foxp3/NFAT interaction measured by alpha screen (Figure 6C). Further experiments are needed to evaluate if this head-to-tail cyclization enhances peptide stability, resistance to protease degradation, and consequently the peptide pharmacokinetic in vivo. 

**Figure 7:**
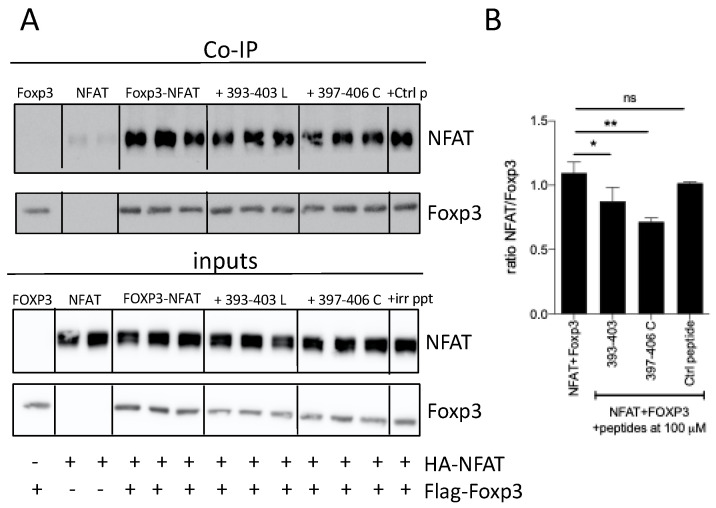
Inhibition of Foxp3–NFAT interaction by peptides 393–403 and 397–406 C. (**A**) HEK293 cells were transfected with Flag-Foxp3 and HA-NFAT; then, they were incubated in the presence of the indicated peptides. After 48 h, cells were lysed with NP-40 lysis buffer. Lysates were incubated with anti-Flag coupled beads and immunoblots were analyzed with antibodies against Flag and HA. (**B**) Densitometric analysis of the Western blot assay * *p* < 0.05, ** *p* < 0.01, one-way ANOVA with Bonferroni multiple comparison test.

To investigate if these peptides were able to impair the association between FOXP3 and NFAT, co-immunoprecipitation experiments were performed on lysates from HEK293 cells ectopically expressing HA-NFAT or Flag-Foxp3. We found that peptide 393–403 and the cyclic version 397–406 C were able to reduce significantly the interaction of between Foxp3 and NFAT (Figure 7). 

## 4. Discussion

The expression of FOXP3 transcription factor and its capacity to interact with other proteins is essential for the immunosuppressive activity of Treg cells. Transcription factors such as FOXP3 are challenging targets for current drug modalities, but it is clear that those molecules are able to inhibit a particular interaction between FOXP3 and its partners and modify the FOXP3 interactome might have an impact on Treg activity. FOXP3 is a complex transcription factor that includes several domains performing different and sometimes opposing functions affecting cancer growth. Indeed, in addition to its clear role of FOXP3 in the immunosuppressive activity of Tregs, its expression in normal non-hematopoietic cells as well as in some cancer cells has been shown to participate in tumor growth control (reviewed in [46]). FOXP3 signaling pathway and genetic and/or epigenetic inactivation of the FOXP3 has been shown to contribute to the malignant transformation of cells [29]. FOXP3 may behave as a transcriptional repressor of SKP2 and HER2, acting as a potential tumor-suppressor gene in breast cancer [47,48], prostate [49], gastric cancer [50], or hepatocellular carcinoma [51]. In our hands, transfection of the FOXP3 gene into 4T1 cells impaired their proliferative capacity. We have not demonstrated formally that ectopic FOXP3 expression is exerting a direct tumor-suppressive activity on 4T1 tumor cells. Our assumption is based on previous works showing this effect on hepatocarcinoma, ovarian, melanoma, or breast cancer cells [32,47,48,52,53]. Interestingly, tumor suppressor and T-regulatory functions of FOXP3 seem to be mediated through separate signaling pathways. It has been described that the tumor-suppressor activity of FOXP3 is mediated by the N-terminal region of the protein [31,51]. In this scenario, it is reasonable to propose that the search of molecules to inhibit FOXP3 immunosuppressive activity should avoid altering the interactions of its N-terminal region harboring the antitumor activity. Phage-displayed peptide libraries allowed us to identify peptides able to bind to unknown parts of FOXP3. Some of them (i.e., p50, p52, and p65) were able to inhibit Treg activity but also impaired its potential antitumor activity, so they were discarded as potential specific and selective inhibitors of the immunosuppressive activity of Tregs. It could be speculated that these peptides could bind to the N-terminal part implicated in the tumor-suppressor activity of FOXP3. However, additional experiments are needed to confirm this hypothesis. These data may indicate that in the case of multifaceted transcription factors such as FOXP3, with different and opposed functional effects, it is important to fine-tune the design of potential inhibitors targeting the domain responsible for the function to be inhibited.

Further characterization of the P60 peptide, one of the Treg inhibitors that does not alter the antitumor activity of FOXP3, allowed us to demonstrate its binding capacity to the intermediate region of FOXP3, impairing FOXP3 dimerization, FOXP3/AML1 interaction, as well as FOXP3 nuclear translocation. A P60 peptide optimization program including alanine scanning, the introduction of D-aminoacids, or head-to-tail cyclization allowed us to improve its stability and efficacy in vitro and in vivo [23]. In this work, we have demonstrated that the P60-derived macrocyclic peptide CM1315 is able to impair tumor progression in two different murine tumor models, opening the door for a next step to advance this compound toward its clinical application. 

In addition to our work with P60 peptide and its derivates, we have identified synthetic peptides that are able to bind the intermediate and the C-terminal parts of the FOXP3 protein. In this approach, we used 15-mer overlapping peptides encompassing FOXP3 sequences as a potential tool to identify decoy molecules that could inhibit FOXP3 dimerization or impair FOXP3 interactome. Thus, we identified the peptide 250–265 corresponding to the LZ that inhibits Treg activity in vitro. Previous reports have identified aminoacids K, E, and K (250–252) as key residues in FOXP3 dimerization [33,34], so it is tempting to postulate that peptide F250–265 could inhibit Treg activity by impairing FOXP3 dimer formation. We also found that peptide F304–318 was able to inhibit Treg activity in vitro. Since mutations at positions close to aminoacid 329 have been shown to impair FOXP3/AML1 interaction and alter Treg suppressive activity [35], we could speculate that peptide F304–318 could affect this protein–protein interaction. However, further experiments are needed to identify the mechanism of action of this peptide. 

We also searched potential FOXP3 inhibitors by using overlapping peptides encompassing the FKH domain, which is responsible for DNA binding and consequently for its activator/repressor functions. An important number of patients with IPEX syndrome have missense mutations in exons encoding the FKH domain [54,55,56,57,58]. FOXP3 requires multimerization (with FOXP3 and with more than other 350 proteins [17]) to exert its functions. Thus, it seems that both the DNA-binding FKH domain and the leucine-zipper domain of FOXP3 are needed for multimerization of FOXP3, DNA binding, and consequently for FOXP3-mediated suppressor functions [37,59]. Thus, those peptides that are able to bind to the FKH domain of FOXP3 might potentially alter the FOXP3 dimerization and consequently the Treg suppressor activity. However, also, peptides derived from the FKH domain could act as FKH decoy molecules and compete for binding to FKH partners. Both approaches could result in potential Treg inhibitors. We identified peptides F363–377, F368–382, F378–392, and F383–397 as good binders to FOXP3. These peptides define an intermediate region within the FKH domain (Figure 4B), which includes the FOXP3 FKH dimerization interface [37]. Notably, two mutations in this interface, F371C and F373A, have been described in IPEX patients, [38,47], suggesting that inhibition of the FKH dimerization process may affect Treg activity. Unfortunately, two of the best peptide binders identified by SPR (peptides F363–377, F368–382) presented some toxicity in T-cell co-cultures, and we could not evaluate their potential Treg inhibitory activity. 

We focused on a peptide encompassing aminoacids 393–403, which correspond to a region implicated in FOXP/NFAT interaction, which is also key for Treg suppressive activity. FOXP3/NFAT interaction can regulate NFAT activity by (i) competing for binding to DNA [60,61], (ii) by sequestering NFAT or other NFAT partners [62,63], or (iii) by forming a cooperative complex [18,64]. The cooperative complex between FOXP3 and NFAT is required to repress the expression of the cytokine IL2, upregulate the expression of the Treg markers CTLA4 and CD25, and confer suppressor function [37,64]. In a previous work, we found that the disruption of FOXP3/NFAT interaction with peptide F393–403 enhanced T-cell proliferation and the production of cytokines IL-2, IFN-γ, or IL-17 in response to TCR stimulation. Moreover, and despite the poor pharmacokinetics of peptides, an in vivo administration of peptide F393–403 exerted antitumor activity in murine tumor models [12]. In this work, we tried to improve the efficacy of the peptide by improving its cell permeability. Conjugation of the F393–403 peptide with the cationic cell-penetrating peptide TAT significantly improved the Treg suppressive activity in vitro. Cell-permeable peptides represent an emerging class of therapeutic molecules, especially for targeting intracellular protein–protein interactions. Further experiments are needed to evaluate if this improvement in cell permeability might also enhance the antitumor activity of the active peptide F393–403 in vivo. We also tried to improve peptide stability by using head-to-tail cyclization. Based on the crystal structure of the FOXP3 FKH domain, and in an attempt to reduce the size of the active peptide, we focused on peptide F397–406, which was synthesized as a head-to-tail cyclic version. This modification improved the inhibitory capacity. New experiments are now needed to evaluate if this cyclization improves its cellular uptake, metabolic stability, and the pharmacokinetics of the peptide.

## 5. Conclusions

**Figure 8:**
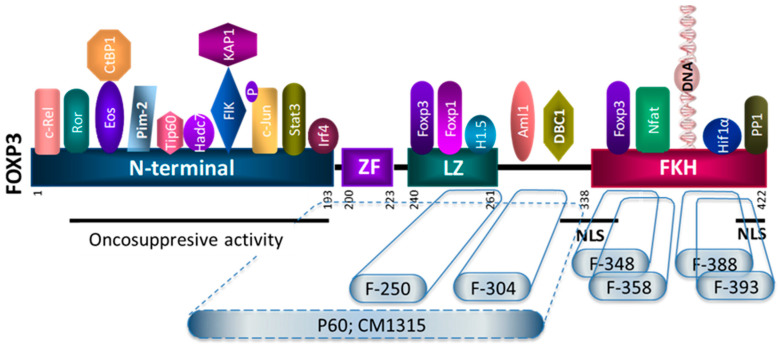
Peptide inhibitors of T regulatory cell activity targeting specific domains of the FOXP3 transcription factor. Schematic representation of some of the FOXP3 interactome and their site of interactions (reviewed in [46]). Peptides identified in this study as potential Treg inhibitors are depicted. F-250, F-304, F-348, F-358, F-388, and F-399 correspond to linear peptides encompassing 15 aminoacids from the FOXP3 sequence. The number correspond to the first aminoacid of the 15-mer peptide. P60 and CM1315 peptides have been derived from a phage-displayed peptide library, and it is expected to bind to the intermediate region of FOXP3 ([23]. ZF: Zinc finger; LZ: leuzin zipper; FKH: forkhead domain; NLS: nuclear localization sequences.

The development of FOXP3 inhibitors for cancer treatment should be able to impair the immunoregulatory functions of FOXP3 on T-cells without altering the anti-proliferative activity that FOXP3 might play in tumor cells. Through a screening campaign using phage displayed peptide libraries and short overlapping peptides encompassing the intermediate and the C terminal part of FOXP3, we identified a panel of Treg inhibitors with relevant Treg inhibitory capacity. Despite the limitations and the need for further confirmatory studies, this work allowed us to search the Achilles heel of FOXP3 and identify short sequences within the leucin zipper, the AML1 binding region, or the FKH domain that could be considered as potential hits (Figure 8) for the development of first-in-class molecules to inhibit FOXP3 with potential for the development of new therapies against cancer.

## 6. Patents

A patent application has been filed on P60 and related compounds. 

## Figures and Tables

**Table 1:** Peptide sequences derived from phage-displayed peptide library screening.

SEQ ID Ner:	Peptide Sequence
p45	RDGKWGSWRGRLMAP
p46	PKAFQYGGRAVGGLW
p47	GMRFFPWLGVGFAMR
p48	ARATFYFGGIVTSKV
p49	GLRERMRLPFFVFGG
p50	IRGLRFGPGFMWPTL
p51	RFRGLISLSQVYLSP
p52	RRQIHLVLPWRAVQS
p53	RSRFFAPFAFLSSGL
p54	LAFRPSSFFARLAYL
p55	SSKSLAAPLGLFVVG
p56	GRVSFSFVAHTWSSV
p57	ADLFLLFLDAVGRSG
p58	GMRFFPWLGVGFAMR
p59	RFWDYDLMLRVLRPL
p60	RDFQSFRKMWPFFAM
p61	RRIVSQLLHPLWSMP
p62	PLFTWSSSRFLRPGS
p63	PGNRLPLPARSFTRS
p64	AWAHSVDAILYLAGS
p65	GGFSLHPWWRFNHDR
p66	QRREAFLHSVLSKFG
p67	FRWVPKFFSAAALPR
p68	GGVHKHSPVGRVRIE
p69	GLSLLYRLSHGFRGV
Ctrl peptide (Ova 254–267)	QLESIINFEKLTEV

**Table 2:** Peptide sequences derived from FOXP3 protein.

Foxp3 Domain	SEQ ID Ner:	Peptide Sequence
**Leuzin zipper domain**	**F245**	QLVLEKEKLSAMQAH
	**F250**	KEKLSAMQAHLAGKM
	**F255**	AMQAHLAGKMALTKA
**AML1 binding domain**	**F282**	IVAAGSQGPVVPAWS
	**F287**	QGPVVPAWSGPREAP
	**F299**	REAPDSLFAVRRHLW
	**F304**	SLFAVRRHLWGSHGN
	**F309**	RRHLWGSHGNSTFPE
	**F323**	FHNMRPPFTYATLIR
**FKH domain**	**F338**	PPFTYATLIRWAILE
	**F343**	ATLIRWAILEAPEKQ
	**F348**	WAILEAPEKQRTLNE
	**F353**	APEKQRTLNEIYHWF
	**F358**	RTLNEIYHWFTRMFA
	**F363**	IYHWFTRMFAFFRNH
	**F368**	TRMFAFFRNHPATWK
	**F373**	FFRNHPATWKNAIRH
	**F378**	PATWKNAIRHNLSLH
	**F383**	NAIRHNLSLHKCFVR
	**F388**	NLSLHKCFVRVESEK
	**F393**	KCFVRVESEKGAVWT
	**F393–403**	KCFVRVESEKG
	**F397–406**	RVESEKGAVW
	**TAT-393–493**	CGISYGRKKRRQRRR-KCFVRVESEKG
**Ctrl peptide (Ova 254–267)**		QLESIINFEKLTEV

## Data Availability

No new data were created or analyzed in this study. Data sharing is not applicable to this article.
